# The efficacy of managing fluid overload in chronic peritoneal dialysis patients by a structured nurse-led intervention protocol

**DOI:** 10.1186/s12882-019-1596-3

**Published:** 2019-12-09

**Authors:** Man Ching Law, Bonnie Ching-Ha Kwan, Janny Suk-Fun Fung, Kai Ming Chow, Jack K.C. Ng, Wing-Fai Pang, Phyllis Mei-Shan Cheng, Chi Bon Leung, Philip Kam-Tao Li, Cheuk Chun Szeto

**Affiliations:** Carol and Richard Yu Peritoneal Dialysis Research Centre, Department of Medicine and Therapeutics, Prince of Wales Hospital, The Chinese University of Hong Kong, Shatin, NT, Hong Kong, SAR China

**Keywords:** Peritoneal dialysis, Bio-impedance spectroscopy, Chronic kidney disease, Cardiovascular disease, Hypertension

## Abstract

**Background:**

Extracellular volume overload is a common problem in peritoneal dialysis (PD) patients and is associated with excessive mortality. We determine the effectiveness of treating PD patients with extracellular volume overload by a structured nurse-led intervention program.

**Methods:**

The hydration status of PD patients was screened by bioimpedance spectroscopy (BIS). Fluid overload was defined as overhydration volume ≥ 2 L. Patients were classified into Symptomatic and Asymptomatic Groups and were managed by a structured nurse-led intervention protocol that focused on education and motivation. Hypertonic cycles were given for short term symptom relief for the Symptomatic group. Patients were followed for 12 weeks for the change in volume status, blood pressure, knowledge and adherence as determined by standard questionnaires.

**Results:**

We recruited 103 patients (53 Symptomatic, 50 Asymptomatic Group. There was a significant reduction in overhydration volume 4 weeks after intervention, which was sustained by week 12; the overall reduction in overhydration volume was 0.96 ± 1.43 L at 4 weeks, and 1.06 ± 1.70 L at 12 weeks (*p* < 0.001 for both). The improvement was significant for both Symptomatic and Asymptomatic Groups. There was a concomitant reduction in systolic blood pressure in the Asymptomatic (146.9 ± 20.7 to 136.9 ± 19.5 mmHg, *p* = 0.037) but not Symptomatic group. The scores of knowledge, adherence to dietary control and advices on daily habit at week 4 were all significantly increased, and the improvement was sustained at week 12.

**Conclusions:**

The structured nurse-led intervention protocol has a lasting benefit on the volume status of PD patients with extracellular volume overload. BIS screening allows prompt identification of volume overload in asymptomatic patients, and facilitates a focused effort on this high risk group.

## Background

Fluid overload is a common problem in peritoneal dialysis (PD) patients [1–4]. A number of previous studies showed that fluid overload is associated with left ventricular hypertrophy and other adverse cardiac outcome in dialysis patients [4–10]. Fluid management is therefore an important treatment target in PD patient.

Unfortunately, the diagnosis and monitoring of fluid overload in PD patients has long been relying on the presence of clinical assessment of signs and symptoms. Previous studies, however, found that stable asymptomatic PD patients could have substantial fluid overloaded [11, 12]. In recent years, bioimpedance spectroscopy is increasingly used as an objective assessment tool for the hydration status of dialysis patients. By quantifying the degree of overhydration (OH), bioimpedance spectroscopy can be used as a guide to the management of fluid status in dialysis patients [13–16]. In PD patients, the index of OH has been shown to correlate with left ventricular mass, blood pressure, and probably patient survival rate [17]. Bioimpedance spectroscopy has the potential of identifying asymptomatic dialysis patients with fluid overloaded and allow timely intervention.

Renal nurse plays an important role in the management of PD patients [18, 19]. In Hong Kong, PD patients with common problems related to dialysis are assessed and managed in the renal nurse clinic by pre-approved intervention protocols [20, 21]. Although nurse-led intervention protocols have been reported to improve the dietary adherence and quality of life of PD patients [20, 22], the efficacy of this approach for volume control of fluid overloaded patients remains undetermined. The objective of this study is to determine the effectiveness of treating PD patients with fluid overload by a structured nurse-led intervention protocol.

## Methods

The study was approved by The Joint Chinese University of Hong Kong – New Territories East Cluster Clinical Research Ethics Committee (reference number CREC-2013.268). All study procedures were in compliance with the Declaration of Helsinki. The study was registered at ClinicalTrials.gov (registration number NCT02168283).

### Patient selection

The hydration status of prevalent PD patients in a single PD centre from October 2013 to September 2015 was screened by bioimpedance spectroscopy. Patients with overhydration (OH) ≥2 L were eligible to join the study. The choice of OH ≥2 L as the cut off was based on our in-house data, which showed that OH above this level had increased blood pressure and need of admission for fluid problem [23]. We excluded patients who were in overt pulmonary oedema and required urgent medical care, who had cognitive impairment or problem of communication, who were unlikely to survive for more than three months, had mechanical problems of the dialysis catheter, had active peritonitis or peritoneal failure. Recruited patients were classified into two groups: patients with clinical features of fluid overload (e.g. dyspnoea on exertion, peripheral oedema, pulmonary congestion) (Symptomatic Group), patients who were clinically asymptomatic (Asymptomatic Group).

### Nurse-led intervention

Written informed consent was obtained. The patients were assessed and managed by a renal nurse specialist in the nurse clinic according to a standardized protocol approved by the Hong Kong Hospital Authority. Briefly, the management procedures include clinical assessment, review of dialysis record, extra hypertonic cycles (4.25% 2 L PD fluid every two hours for two cycles in Symptomatic Group) immediately, modification of home PD regimen by changing the patient’s regular PD fluid regimen from 1.5 to 2.5% in one of the bag-exchanges daily for three to five days or till next assessment in Symptomatic Group, dietary counselling on fluid and salt restriction, and other relevant lifestyle modification advice. Dietary counselling includes one-week dietary record reviewing, identification of undesirable food with suggestions on alternatives to avoid excess salt and fluid intake. Lifestyle modification advices include identification of daily habit that would lead to excess salt and fluid intake, tailor-made plans with patient in changing the identified unwanted habit, and promoting a sustained lifestyle modification. All counselling sessions were conducted by nurse specialists who were equipped with adult learning knowledge and patient motivation skills. Patients were referred to nephrologists for further assessment and treatment if there were features of clinical instability or problems unrelated to simple fluid overload. Depending on the rate of clinical improvement, the PD regimen of all patients was switched back to the baseline one within 2 weeks, and the dosage of diuretic therapy was not changed during the study period.

### Bioimpedance spectroscopy

The Body Composition Monitor (Fresenius Medical Care, Germany) was used for bioimpedance spectroscopy study to measure the fluid status at baseline and then 4 and 12 weeks after treatment. The method of bioimpedance spectroscopy was described previously [23]. Briefly, electrodes were attached to one hand and one foot with the patient in a supine position. After patient cable was connected, the measurement would complete automatically in 2 min. We computed the following parameters from this test: total body water (TBW), intracellular water (ICW), and extracellular water (ECW), lean tissue mass, adipose tissue mass, and volume of overhydration (OH).

### Assessment of patient knowledge and adherence

We assessed the adherence to salt and fluid restriction, and to lifestyle modification advices at 0, 4 and 12 weeks. The questionnaire is listed in Additional file 1. For the adherence to dietary advices, a standard questionnaire was designed with a list of common local food items was used. Similarly, for the adherence to lifestyle modification advices, a standard questionnaire with a list of 10 usual daily habits was used. Patients were asked to identify undesirable food items and daily habits in the two questionnaires within the past week and the respective adherence score was computed. For the assessment of patient knowledge, a standard questionnaire was designed to focus on the concept on salt and fluid restriction, undesirable effects of fluid overload, and benefits of good fluid control in PD patients (Additional file 2). All three questionnaires were designed in-house and vetted by three nursing specialists and dietitians.

### Follow-up assessment and outcome measures

After baseline assessment and treatment, the fluid status of all patients were reassessed after 4 days, 4 weeks, and 12 weeks by a renal nurse specialist. Bioimpedance spectroscopy was repeated at 4 and 12 weeks. The primary outcome was the volume of overhydration (OH) at follow up visits. Secondary outcome measures included blood pressure, as well as the knowledge on salt and fluid restriction.

### Sample size justification

The sample size was estimated by the Power Analysis and Sample Size for Windows software (PASS 2000, NCSS, Kaysville, Utah). Our in-house data showed that the standard deviation of overhydration of PD patient is 2 L. We assumed a reduction of overhydration by 1 L to be clinically meaningful. A sample size of 45 patients would achieve 90% power to detect such a reduction in overhydration at a significance level of 0.05, using a two-sided paired Student’s t test.

### Statistical analysis

Statistical analysis was performed by SPSS for Windows software version 15.0 (SPSS Inc., Chicago, IL). Descriptive data was represented as mean ± SD. Data were compared by paired Student’s t test or analysis of variance (ANOVA) for repeated measurements as appropriate. Correlation between continuous variables would be explored by Pearson’s correlation coefficient. A *p*-value of less than 0.05 was considered significant. All probabilities were two-tailed.

## Results

**Fig. 1:**
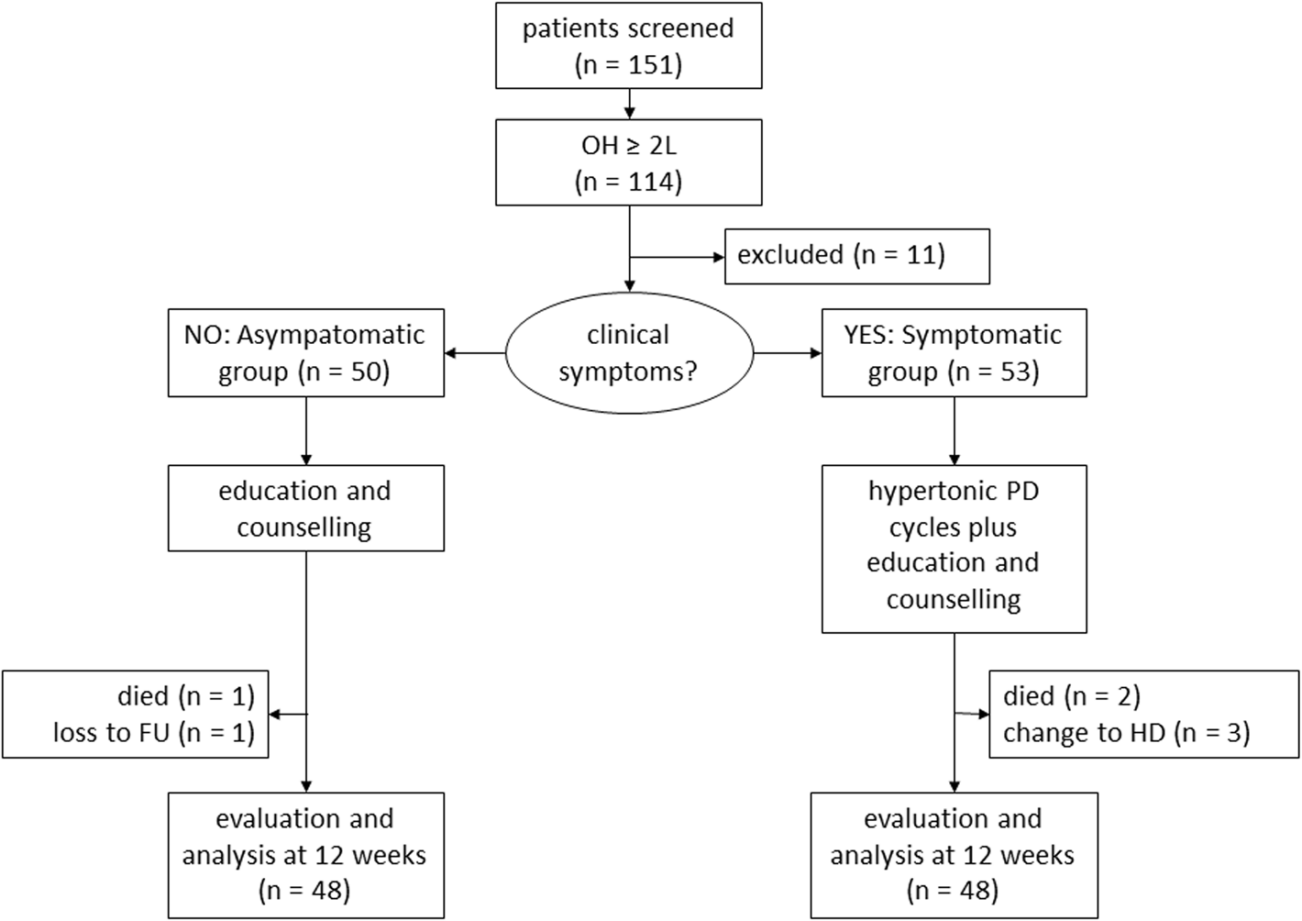
Summary of study flow. (OH, overhydration; FU, follow up; PD, peritoneal dialysis; HD, hemodialysis; 11 patients were excluded for clinical instability and were referred to nephrologists for medical care)

We screened 151 PD patients; 114 were eligible for the study. In 103 of them, consent was obtained; another 11 patients were excluded because of rapid progression of symptoms and were referred to nephrologists. Figure 1 shows the study flow and patient recruitment process. Amongst the 103 PD patients 92 were on continuous ambulatory peritoneal dialysis (CAPD), and 11 on machine assisted PD. After 12 weeks, 96 completed the study. One patient from each group died of chest infection; another patient in the symptomatic group died of myocardial infarction. Baseline demographic and clinical data are summarized in Table 1. Baseline laboratory and bioimpedance spectroscopy data are summarized in Table 2. There is no significant correlation between baseline blood pressure and volume status of the patients (details not shown).

**Table 1:** Baseline clinical and demographic data

	all patients	Symptomatic	Asymptomatic
No. of patients	96	48	48
Sex (M:F)	56:40	25:23	31:17
Age (year)	61.1 ± 9.0	61.2 ± 9.8	61.0 ± 8.2
Duration of dialysis (months)	17.4 ± 23.4	19.1 ± 24.7	15.6 ± 22.2
Body height (cm)	162.1 ± 6.6	162.3 ± 6.3	161.9 ± 7.0
Body weight (kg)	65.5 ± 10.8	69.2 ± 9.9	61.8 ± 10.5
Body mass index (kg/m^2^)	24.9 ± 3.8	26.3 ± 3.6	23.6 ± 3.6
Blood pressure (mmHg)			
Systolic	146 ± 21	146 ± 22	146 ± 20
Diastolic	75 ± 12	74 ± 12	76 ± 13
Causes of renal failure, no. of cases (%)			
Diabetic nephropathy	54 (56.3%)	31 (64.6%)	23 (47.9%)
Glomerulonephritis	15 (15.6%)	5 (10.4%)	10 (20.8%)
Hypertensive nephrosclerosis	7 (7.3%)	3 (6.3%)	4 (8.3%)
Polycystic kidney	5 (5.2%)	4 (8.3%)	1 (2.1%)
Obstructive uropathy	0	0	0
Others / unknown	15 (15.6%)	5 (10.4%)	10 (20.8%)
Comorbidities, no. of cases (%)			
Diabetes	67 (69.8%)	34 (70.8%)	33 (68.8%)
Ischemic heart disease	20 (20.8%)	9 (18.8%)	11 (22.9%)
Cerebrovascular disease	13 (13.5%)	10 (20.8%)	3 (6.3%)
Charlson’s Comorbidity Index	6.1 ± 2.2	6.4 ± 2.2	6.0 ± 2.1

**Table 2:** Baseline biochemical and bioimpedance spectroscopy parameters

	all patients	Symptomatic	Asymptomatic
No. of patients	96	48	48
hemoglobin (g/dL)	9.3 ± 1.4	9.3 ± 1.4	9.4 ± 1.5
serum albumin (g/L)	34.4 ± 4.5	34.4 ± 4.3	34.4 ± 4.7
peritoneal transport			
D/P creatinine at 4 h	0.67 ± 0.14	0.66 ± 0.15	0.68 ± 0.14
MTAC creatinine (ml/min/1.73m^2^)	10.94 ± 6.14	10.80 ± 7.44	11.08 ± 4.64
residual GFR (ml/min/1.73m^2^)	2.69 ± 3.09	2.03 ± 2.75	3.16 ± 3.26
urine output (L/day)	0.732 ± 0.772	0.690 ± 0.892	0.761 ± 0.688
frusemide (mg/day)	79.2 ± 103.9	53.1 ± 88.3	105.2 ± 112.5
weekly Kt/V	1.91 ± 0.59	1.82 ± 0.66	2.0 ± 0.53
ultrafiltration volume (L/day)	0.487 ± 0.650	0.388 ± 0.773	0.584 ± 0.490
bioimpedance spectroscopy			
LTI (kg/m^2^)	14.2 ± 3.0	14.7 ± 3.0	13.8 ± 2.9
FTI (kg/m^2^)	8.6 ± 3.4	9.0 ± 3.7	8.3 ± 3.1
OH (L)	4.9 ± 2.2	6.0 ± 2.3	3.8 ± 1.4
TBW (L)	36.8 ± 6.6	39.0 ± 6.5	34.5 ± 6.0
ECW (L)	19.1 ± 3.6	20.7 ± 3.4	17.5 ± 2.9
ICW (L)	17.7 ± 3.5	18.3 ± 3.5	17.1 ± 3.4
ECW/ICW	1.09 ± 0.14	1.14 ± 0.14	1.03 ± 0.11

*D/P* dialysate-to-plasma concentration ratio, *MTAC* mass transfer area coefficient, *LTI* lean tissue index, *FTI* fat tissue index, *OH* overhydration, *TBW* total body water, *ECW* extracellular water, *ICW* intracellular water

### Improvement of fluid overload and blood pressure control

**Fig. 2:**
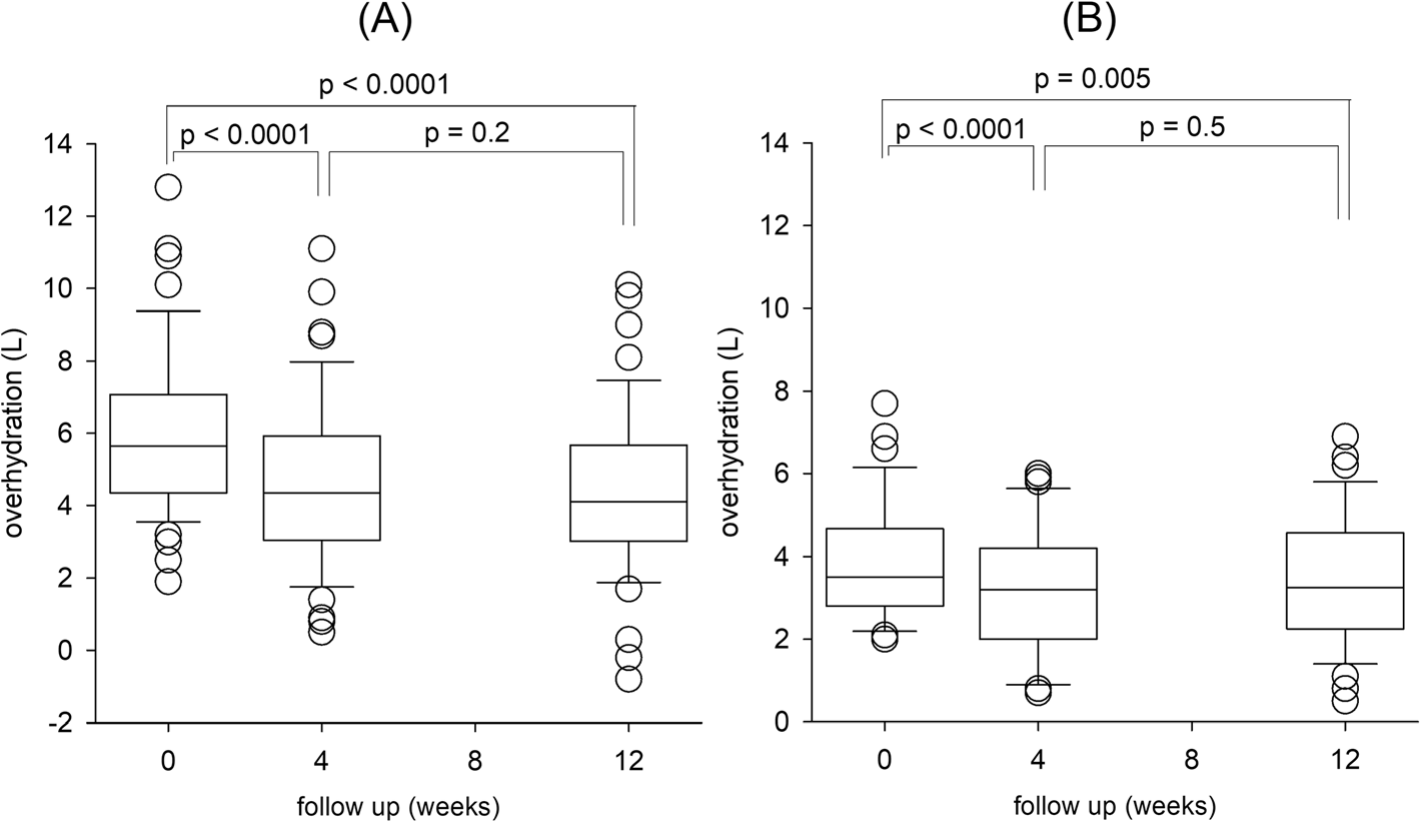
Change in overhydration volume during the study period in (A) Symptomatic; and (B) Asymptomatic Group. (Whisker-box plots, with boxes indicate median, 25th and 75th percentiles, whiskers indicate 5th and 95th percentiles. Data are compared by paired Student’s t test)

The changes in hydration status and bioimpedance spectroscopic parameters during follow up are summarized in Table 3. We found a significant reduction in overhydration volume 4 weeks after intervention, and the improvement was sustained by week 12, and the reduction in overhydration was significant for both Symptomatic and Asymptomatic Groups (Fig. 2). The overall reduction in overhydration volume was 0.96 ± 1.43 L at 4 weeks, and 1.06 ± 1.70 L at 12 weeks. There was also a significant reduction in ECW/ICW ratio in both Symptomatic and Asymptomatic Groups four weeks after intervention. On the other hand, improvement in TBW and ECW volumes was significant in Symptomatic group only. The reduction in OH volume was significantly more in the Symptomatic than Asymptomatic Group from baseline to week 4 (− 1.28 ± 1.69 L vs − 0.64 ± 1.01 L, *p* = 0.026) and to week 12 (− 1.60 ± 1.96 L vs − 0.51 ± 1.19 L, *p* = 0.001).

**Table 3:** Change in bioimpedance spectroscopy parameters during the study

	all patients			Symptomatic			Asymptomatic		
	Week 0	Week 4	Week 12	Week 0	Week 4	Week 12	Week 0	Week 4	Week 12
OH (L)	4.9 ± 2.2	4.0 ± 2.1*	3.9 ± 2.0*	6.0 ± 2.3	4.7 ± 2.3*	4.4 ± 2.3*	3.9 ± 1.4	3.2 ± 1.5*	3.4 ± 1.6*
TBW (L)	37.2 ± 6.1	36.1 ± 6.4*	36.1 ± 6.2*	38.6 ± 6.3	37.0 ± 6.7*	36.6 ± 6.4*	35.4 ± 5.4	35.0 ± 5.8	35.4 ± 5.9
ECW (L)	19.2 ± 3.2	18.2 ± 3.3*	18.2 ± 3.2*	20.3 ± 3.2	19.0 ± 3.5*	18.8 ± 3.4*	17.8 ± 2.7	17.2 ± 2.8	17.5 ± 2.9
ICW (L)	18.0 ± 3.3	17.9 ± 3.5	17.9 ± 3.3	18.3 ± 3.5	18.0 ± 3.7	17.9 ± 3.4	17.6 ± 3.0	17.7 ± 3.2	18.0 ± 3.3
ECW/ICW	1.08 ± 0.13	1.03 ± 0.13*	1.02 ± 0.13*	1.13 ± 0.13	1.07 ± 0.14*	1.06 ± 0.13*	1.02 ± 0.09	0.98 ± 0.11*	0.98 ± 0.11*
LTI (kg/m^2^)	14.4 ± 2.8	14.3 ± 3.0	14.4 ± 2.8	14.6 ± 3.0	14.4 ± 3.1	14.3 ± 2.9	14.1 ± 2.7	14.3 ± 2.9	14.5 ± 2.7
FTI (kg/m^2^)	8.6 ± 3.5	8.5 ± 3.4	8.7 ± 3.3	9.0 ± 3.7	9.0 ± 3.6	9.3 ± 3.2	8.0 ± 3.1	7.9 ± 3.2	7.8 ± 3.2

*OH* overhydration, *TBW* total body water, *ECW* extracellular water, *ICW* intracellular water, *LTI* lean tissue index, *FTI* fat tissue index

*p < 0.05 when compared with baseline

**Fig. 3:**
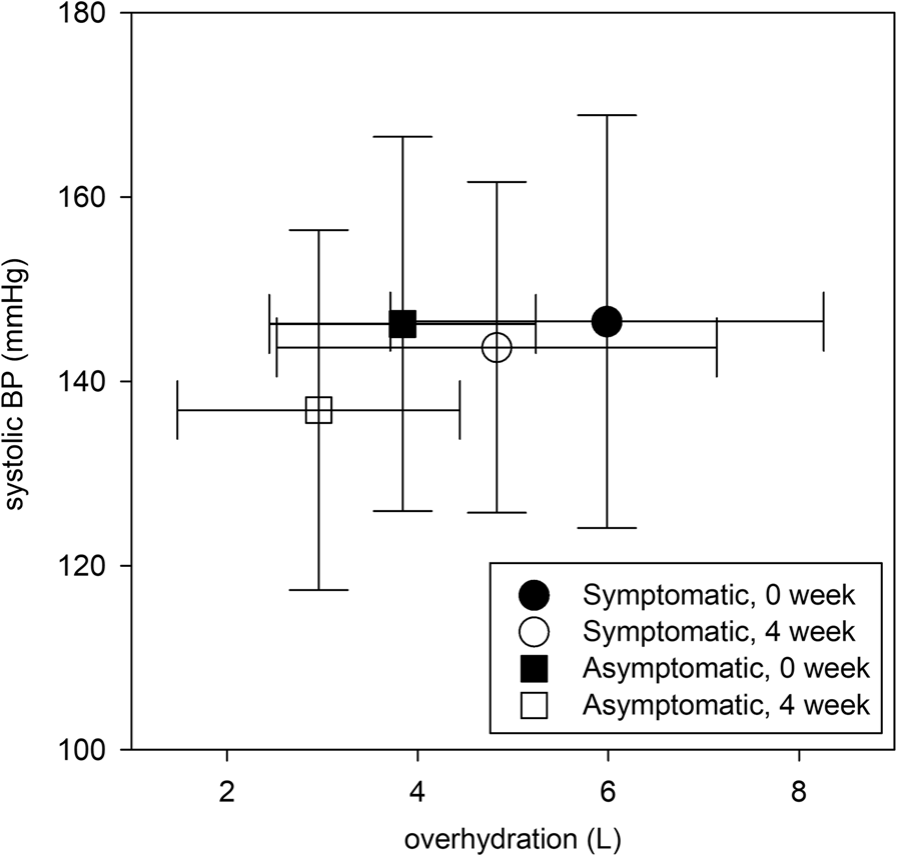
Relation between overhydration volume and systolic blood pressure. Error bars denote standard deviations

In addition to improvement in overhydration volume, there was a concomitant reduction in systolic, but not diastolic, blood pressure. The overall reduction in systolic blood pressure was 5.63 ± 25.35 mmHg at 4 weeks, and 3.22 ± 24.83 mmHg at 12 weeks. Subgroup analysis showed that systolic blood pressure was significantly reduced only in Asymptomatic Group (146.9 ± 20.7 to 136.9 ± 19.5 mmHg, *p* = 0.037) but not the Symptomatic group (145.6 ± 22.6 to 143.7 ± 18.0 mmHg, *p* = 0.6) (Fig. 3). There was no significant correlation between the reduction in overhydration volume and systolic blood pressure (r = 0.160, *p* = 0.15). Body weight of the Symptomatic Group reduced from 69.2 ± 9.9 kg at baseline to 66.8 ± 9.8 kg at 4 weeks, and 67.3 ± 9.8 kg at 12 weeks (*p* < 0.001 for both). In contrast, there was no significant change in body weight of the Asymptomatic Group, which was 61.8 ± 10.5, 61.1 ± 10.9, and 63.1 ± 10.6 kg at baseline, 4, and 12 weeks respectively (p = 0.15 and *p* = 0.5, respectively).

### Knowledge and adherence

**Fig. 4:**
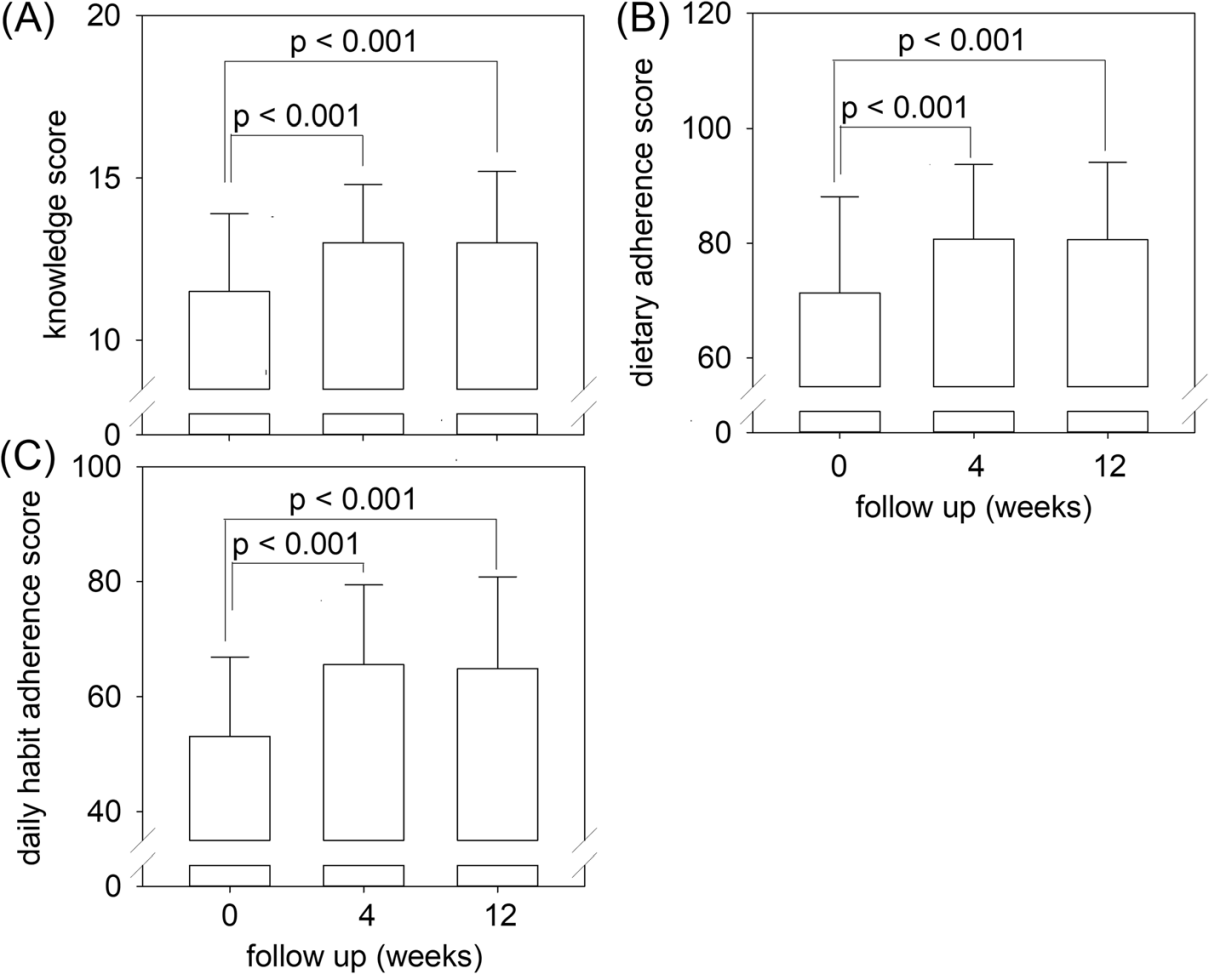
Change in clinical scores during the study: (A) knowledge score related to fluid control; (B) adherence score for salt and fluid restriction; and (C) adherence score to advices on daily habit. (Error bars denote standard deviations. Data are compared by paired Student’s t test)

The scores on patient knowledge related to fluid control (K), patient adherence on dietary control (DC), and patient adherence to advices on daily habit (DH) are summarized in Fig. 4. There was a significant improvement in all three scores at week 4, and the improvement was sustained at week 12. The improvement on all three scores was statistically significant when Symptomatic and Asymptomatic Groups were analysed separately (Table 4).

**Table 4:** Change in patient knowledge and adherence score of the two groups during the study

	Symptomatic			Asymptomatic		
	Week 0	Week 4	Week 12	Week 0	Week 4	Week 12
knowledge related to fluid control	11.5 ± 2.5	13.2 ± 1.8***	13.2 ± 2.1***	11.5 ± 2.5	12.8 ± 1.8**	12.9 ± 2.2**
adherence in salt and fluid restriction	75.3 ± 15.1	82.7 ± 12.4**	83.4 ± 12.0**	67.4 ± 17.6	78.8 ± 13.5***	77.9 ± 14.5***
adherence to advices on daily habit	53.6 ± 14.9	66.6 ± 14.3***	65.1 ± 17.0***	58.5 ± 12.4	64.7 ± 13.5**	64.6 ± 15.0*

*p < 0.05, **p < 0.01, and ***p < 0.001 when compared with baseline

### Utilization of medical services

We further analyze the utilization of medical services on our patients. Of the 96 patients who completed the study, 67 did not require extra medical consultation or unplanned admission during the study period. For the other 29 patients, only 11 required medical attention due to fluid overload, while 18 required extra medical consultation for unrelated medical reasons.

## Discussion

Fluid overload has been increasingly recognized to negatively affect the quality of life and is a strong predictor of mortality in PD patients [24, 25]. This study examined the efficacy of managing fluid overload in chronic PD patient, both symptomatic and asymptomatic ones, by a structured nurse-led intervention program in an outpatient setting.

We identified asymptomatic overhydrated PD patients by routine bioimpedance spectroscopy screening. For the asymptomatic group, our intervention protocol does not involve any change in the PD regimen, but mainly education and counselling in order to enhance the patients’ knowledge on fluid overload, dietary adherence and motivate them to have lifestyle modifications in order to improve the fluid status. The renal nurse specialist provided intensive counselling to the patients. On average, an one hour counselling was given at the baseline visit, and then two further 15 to 30 min sessions at 4 and 12 weeks. Our results indicate that routine bioimpedance spectroscopy screening of asymptomatic PD patients may facilitate timely interventions in asymptomatic overhydrated PD patients and minimize the use of hypertonic PD cycles. Our result is in line with the findings of several previous studies, which showed that the use of bioimpedance spectroscopy facilitates the clinical management and decision on fluid control for dialysis patients [12, 26, 27].

In this study, we find a significant decrease in overhydration volume at the 4 and 12 weeks in both Symptomatic and Asymptomatic Groups. For the symptomatic group, it was not possible to differentiate whether regimen intensification or counselling was the main factor that led to improvement. In future studies, it would be important to include a control group whose peritoneal ultrafiltration is intensified but without patient counselling. In the asymptomatic group, volume status improved despite hypertonic PD cycles were not used, but the improvement was small and may not be clinically important. The improvement in adhering to dietary advice and adopting appropriate lifestyle modifications is the most probable explanation for the improvement, and the observation is consistent with previous reports. For example, a previous study showed that patient education and counselling by nurses improved dietary adherence in diabetic PD patients, resulting in the improvement in fluid status without the use of hypertonic PD cycles [28]. Another study also showed that interventions that target motivational issues, assess and improve patient knowledge, enhance social support, and facilitate accurate self-assessment of fluid status effectively improve the adherence to fluid restriction of chronic hemodialysis patients [29]. Our study further supports the notion that patient education and counselling are helpful in correcting fluid overload in PD patients by motivating them for dietary adherence and lifestyle modifications.

In this study, we did not observe any correlation between change in overhydration volume and systolic blood pressure. In the asymptomatic group, however, there was a small but statistically significant reduction in blood pressure. The underlying reason is not entirely clear. It is possible that we did not control for the use of anti-hypertensive treatment vigorously, and many of our patients tended to adjust their drug dosage according to their home blood pressure monitoring. In addition, blood pressure of our patients may be affected by reasons unrelated to hydration status (for example, coexisting cardiovascular diseases). Because of the complexity of antihypertensive regimen, we were not able to perform a meaningful analysis on their alteration during the study period – this is a major limitation of our study.

In addition, both Symptomatic and Asymptomatic Groups had significant improvement in patient knowledge, adherence to dietary control and lifestyle advices in the study period. Our result further supports the notion that the benefit of hypertonic PD cycles, which was only used in the Symptomatic group, is mainly for acute symptom relief, while medium to long term improvement in the fluid status relies on the enhancement of patient knowledge and adherence.

For the symptomatic group of this study, our protocol includes the initiation of hypertonic PD cycles by renal nurse specialist on top of standard education and counselling. Unfortunately, it was not our original objective and early symptom change was not assessed in this study. Nonetheless, patients with fluid overload symptoms improved along the 12 weeks study period as indicated by decreasing oedema from physical assessment and body weight measurement, and also indicated by bio-impedance spectroscopy readings. Our observation indicates that PD patients with symptomatic fluid overload could be effectively managed by a protocol-driven nurse-led program, which would reduce the workload of medical staff by avoiding unplanned medical consultations or unplanned admission for the patients. Further studies are needed, however, to determine the optimal criteria for patient triage and management protocol.

There are several limitations in this study. First, this study is not a randomized control trial and some unidentified factors may bias the results. However, our approach is designed for real life clinical situations and is widely applicable. Second, our study is a single centre study with limited duration of follow up. Our result, for example, does not provide any information on the need or frequency of re-education or assessment for the counselled patients.

In summary, our structured nurse-led intervention protocol has significant and sustained benefit on the hydration status for chronic PD patients with fluid overload. Bioimpedance spectroscopy screening is a useful test that allows prompt identification of volume overload in asymptomatic PD patients, and help to direct the focus of nursing effort to this group of high risk patients. Patient education and counselling improve adherence to dietary and lifestyle advices, and play pivotal roles in the sustained improvement. Our protocol is pragmatic, safe, effective, and facilitates the out-patient management of fluid overloaded PD patients.

## Conclusions

The structured nurse-led intervention protocol has a lasting benefit on the volume status of PD patients with extracellular volume overload. BIS screening allows prompt identification of volume overload in asymptomatic patients, and facilitates a focused effort on this high risk group.

## Supplementary information

**Additional file 1.** Questionnaire on the adherence to dietary advice and life style modification

**Additional file 2.** Questionnaire on patient knowledge

## Data Availability

All study data are available from the corresponding author upon written request.
