# Supplementary material for: The efficacy of managing fluid overload in chronic peritoneal dialysis patients by a structured nurse-led intervention protocol
Source: BMC Nephrol. 2019 Dec 9;20:454. doi: 10.1186/s12882-019-1596-3 (PMC6902497; doi:10.1186/s12882-019-1596-3)
Supplement: Supplementary file 1 — Additional file 1. Questionnaire on the adherence to dietary advice and life style modification [file 12882_2019_1596_MOESM1_ESM.docx]

Additional file 1. Questionnaire on the adherence to dietary advice and life style modification

下列問題是有關過去七天你的飲食習慣

在過去七天，你覺得你在鹽份限制方面做得怎樣？以100分為滿分計算，你會給自己多少分？------------------------------------------------------------------------------------

在過去七天，你覺得你在水份限制方面做得怎樣？以100分為滿分計算，你會給自己多少分？------------------------------------------------------------------------------------

在過去七天，你有沒有進食以下食物（即使只有一次）？

|  | 有 | 沒有 |
| --- | --- | --- |
| 奶茶、好立克、罐裝飲品 |  |  |
| 馬利餅、梳打餅 |  |  |
| 即食麵、杯麵之調味包 |  |  |
| 米飯、白方包 |  |  |
| 熱狗包 |  |  |
| 粉絲 |  |  |
| 麥片/粥 |  |  |
| 生菜、菜心、白菜 |  |  |
| 梅菜、榨菜、冬菜、咸酸菜 |  |  |
| 洋蔥、甘荀 |  |  |
| 罐頭蔬菜/豆 |  |  |
| 提子、車厘子 |  |  |
| 涼果，如：話梅、檸檬、加應子 |  |  |
| 新鮮或雪藏肉類 |  |  |
| 火腿、腸仔 |  |  |
| 新鮮或雪藏海產 |  |  |
| 燒味：如叉燒、燒肉、燒鵝、豉油雞 |  |  |
| 蘋果、橙 |  |  |
| 茶樓點心：如蝦餃、燒賣、叉燒包 |  |  |
| 粉、麵 |  |  |
| 豆豉、腐乳、麵豉 |  |  |
| 薑、蔥、胡椒、芫茜 |  |  |
| 腊味：如腊腸、腊鴨 |  |  |
| 蛋白 |  |  |
| 鹵味：如鹵水蛋、鹵水雞翼 |  |  |
| 新鮮或雪藏雞翼 |  |  |
| 豆漿 |  |  |
| 青瓜、勝瓜、合掌瓜 |  |  |
| 罐頭湯 |  |  |
| 洋蔥、甘荀 |  |  |

過去七天的生活習慣：

|  | 0 | 1 | 2 | 3 | 4 | 5 | 6 | 7 |
| --- | --- | --- | --- | --- | --- | --- | --- | --- |
| 在過去七天，你有多少天有在外間的食店進食？ |  |  |  |  |  |  |  |  |
| 在過去七天，除了正餐外，你有多少天有進食零食？ |  |  |  |  |  |  |  |  |
| 在過去七天，你購買食物時，有多少天有先閱讀營養標籤，選擇鹽份（鈉質）較低的食物？ |  |  |  |  |  |  |  |  |
| 在過去七天，你有多少天有計算整天總去水量（量小便 ＋ 磅洗肚水）？ |  |  |  |  |  |  |  |  |
| 在過去七天，你有多少天以早一日總去水量（量小便 ＋ 磅洗肚水），作為進食流質的上限？ |  |  |  |  |  |  |  |  |
| 在過去七天，你有多少天有量體重？ |  |  |  |  |  |  |  |  |
| 在過去七天，你有多少天有刻意減少流質食物進食量？ |  |  |  |  |  |  |  |  |
| 在過去七天，你有多少天有吃粥 / 麥片？ |  |  |  |  |  |  |  |  |
| 在過去七天，你有多少天有進食「即食」食品？ |  |  |  |  |  |  |  |  |
| 在過去七天，你有多少天有在進餐時，需要額外加鹽、鼓油、茄汁、蠔油、醬油等？ |  |  |  |  |  |  |  |  |
