# Supplementary material for: The efficacy of managing fluid overload in chronic peritoneal dialysis patients by a structured nurse-led intervention protocol
Source: BMC Nephrol. 2019 Dec 9;20:454. doi: 10.1186/s12882-019-1596-3 (PMC6902497; doi:10.1186/s12882-019-1596-3)
Supplement: Supplementary file 2 — Additional file 2. Questionnaire on patient knowledge [file 12882_2019_1596_MOESM2_ESM.docx]

Additional file 2. Questionnaire on patient knowledge

腹膜透析病人體內積水過多知識測試問卷

請選擇正確的答案

| 1 | 如果體內積水過多，我可能會：   1. 肚痛 2. 腳腫、面腫 3. 發燒 4. 我不知道 |
| --- | --- |
| 2 | 如果體內積水過多，體重會：   1. 正常 2. 增加 3. 減少 4. 我不知道 |
| 3 | 如果體內積水過多，血壓會：   1. 正常 2. 減低 3. 升高 4. 我不知道 |
| 4 | 下面那一種食物會引發我體內積水過多：   1. 高蛋白質食物 2. 高鹽份（高鈉質）食物 3. 新鮮肉類 4. 我不知道 |
| 5 | 以下那一個句子是正確的？   1. 我飲多些水, 洗肚水便會抽多些水 2. 每袋洗肚水都有抽水上限, 如果飲水份量超過洗肚水抽水上限, 剩餘的水份便會留在體內, 造成積水. 3. 延遲更換洗肚水, 可以增加抽水量 4. 我不知道 |
| 6 | 體內積水過多，會引起以下的後果:   1. 血糖過高 2. 營養不良 3. 肺積水 4. 我不知道 |
| 7 | 長期體內積水過多，可以令我有以下的後遺症：   1. 疲勞 2. 心臟肥大 3. 貧血 4. 我不知道 |
| 8 | 保持體內水份平衡（沒有積水），對我有甚麼好處？   1. 可以減少換洗肚水的次數 2. 可以減少因積水過多而引起的併發症 3. 可以不必控制飲食 4. 我不知道 |
| 9 | 下面那一句是正確的：   1. 我要飲多些水，小便份量才會增加 2. 我不可以飲太多水，我口渴時可以用茶或水果解渴 3. 我洗肚時的淨出水量和平時一樣，但是小便份量減少了，所以我要減少飲水份量 4. 我不知道 |
| 10 | 下面那些食物會令我吸收過多鹽份：   1. 醃製食物如梅菜、冬菜 2. 鮮檸檬 3. 新鮮蔬菜 4. 我不知道 |
| 11 | 以下那種日常生活習慣會增加鹽份（鈉質）的進食量？   1. 常運動 2. 常出外進食 3. 正常上班 4. 我不知道 |
| 12 | 為避免進食鹽份（鈉質）過多，以下那種調味料要減少使用量？   1. 五香粉 2. 胡椒粉 3. 豉油 4. 我不知道 |

| 13 | 以下那些食物，可以自由進食，不會引致鹽份（鈉質）過高？   1. 燒味 2. 罐頭 3. 新鮮蔬菜 4. 我不知道 |
| --- | --- |
| 14 | 以下那一個句子是錯的？   1. 如果口乾，可以口含檸檬片，減少口乾感覺 2. 如果口乾，可以飲用濃茶解渴 3. 如果口乾，可以嚼香口膠，減少口乾感覺 4. 我不知道 |
| 15 | 以下那一個句子是對的？   1. 計算水份飲用量時，用以「濕口」的一、兩「啖」水不必計算 2. 計算水份飲用量時，應包括所有飲品、水果中的水份和流質食物的總容量 3. 計算水份飲用量時，在外進食時的飲用量很難計算，所以不須包括在內 4. 我不知道 |
